# Synthesis, Characterization, Antimicrobial, DNA Cleavage, and *In Vitro* Cytotoxic Studies of Some Metal Complexes of Schiff Base Ligand Derived from Thiazole and Quinoline Moiety

**DOI:** 10.1155/2014/314963

**Published:** 2014-03-05

**Authors:** Nagesh Gunvanthrao Yernale, Mruthyunjayaswamy Bennikallu Hire Mathada

**Affiliations:** Department of Studies and Research in Chemistry, Gulbarga University, GULBARGA, Karnataka 585 106, India

## Abstract

A novel Schiff base ligand *N*-(4-phenylthiazol-2yl)-2-((2-thiaxo-1,2-dihydroquinolin-3-yl)methylene)hydrazinecarboxamide **(L)** obtained by the condensation of *N*-(4-phenylthiazol-2-yl)hydrazinecarboxamide with 2-thioxo-1,2-dihydroquinoline-3-carbaldehyde and its newly synthesized Cu(II), Co(II), Ni(II), and Zn(II) complexes have been characterized by elemental analysis and various spectral studies like FT-IR, ^1^H NMR, ESI mass, UV-Visible, ESR, TGA/DTA, and powder X-ray diffraction studies. The Schiff base ligand **(L)** behaves as tridentate ONS donor and forms the complexes of type [ML(Cl)_2_] with square pyramidal geometry. The Schiff base ligand **(L)** and its metal complexes have been screened *in vitro* for their antibacterial and antifungal activities by minimum inhibitory concentration (MIC) method. The DNA cleavage activity of ligand and its metal complexes were studied using plasmid DNA pBR322 as a target molecule by gel electrophoresis method. The brine shrimp bioassay was also carried out to study the *in vitro* cytotoxicity properties for the ligand and its metal complexes against *Artemia salina*. The results showed that the biological activities of the ligand were found to be increased on complexation.

## 1. Introduction

Schiff bases are important classes of ligands that coordinate with metal ions *via* azomethine nitrogen and have been studied extensively because of increasing recognition of their role in biological system [1]. The Schiff bases containing ONS donor atoms act as superior chelating agents for the transition and nontransition metal ions and showed remarkable biological activities [2, 3]. Coordination of these compounds with metal ions, such as copper and nickel, often enhance their activities [4]. Bonding between azomethine nitrogen and metal ion was found to be important for biological activity. Several azomethines were reported to possess important antibacterial [5], antifungal [6], anticancer [7], and diuretic activities [8].

Thiazoles are one of the most important classes of heterocycles that have attracted a great deal of interest owing to their wide range of biological properties such as antiprotozoal [9], antimicrobial [10], anti-inflammatory [11], CNS depressant [12], antitubercular [13], antitumor [14, 15], anthelmintic [16, 17], antidiabetics [18], and herbicidal [19, 20] activities. Thus, thiazole nucleus has been much studied in the field of medicinal chemistry. Quinolines are a class of nitrogen heterocycles, present in large number of natural and synthetic compounds which exhibit strong biological activities such as antibacterial [21], antifungal [22], antiamoebic [23], antileishmanial [24], antimalarial [25], antitumor [26], immunosuppressive [27], analgesic, vasorelaxing [28], antiplasmodial [29], anticonvulsant, and antihypertensive [30] activities.

A lot of work has been done on the synthesis of compounds using substituted thiazole and quinoline moieties [31, 32]. Literature survey reveals that not much work has been carried out on Schiff base ligand derived from 2-thioxo-1, 2-dihydroquinoline-3-carbaldehyde. In view of the above findings and in continuation of our research work on synthesis and characterization of new Schiff base ligands and their metal complexes, we hereby report the synthesis of a novel Schiff base ligand *N*-(4-phenylthiazol-2yl)-2-((2-thiaxo-1,2-dihydroquinolin-3-yl)methylene) hydrazine carboxamide **(L)** and its Cu(II), Co(II), Ni(II), and Zn(II) complexes, their characterization by various spectroscopic techniques, and their antimicrobial, DNA cleavage, and *in vitro* cytotoxicity property.

## 2. Materials and Methods

### 2.1. Reagents and Instruments

Elemental analysis (C, H, and N) was performed on a Vario EL III CHNS analyzer. IR spectra were recorded on a Perkin Elmer-Spectrum RX-I FTIR spectrophotometer using KBr disc technique in the 4000 to 250 cm^−1^ region. ^1^H NMR spectra were recorded on Bruker Avance II 400 MHz NMR Spectrometer in *d*_6_-DMSO using TMS as an internal standard. ESI mass spectra were recorded by electrospray ionization (ESI) on a Waters Micromass Q-Tof Micro spectrometer. Electronic spectra of the Cu(II), Co(II), and Ni(II) complexes were recorded at 25°C on a Elico-SL 164 double beam spectrometer in quartz cells in the range 200–1000 nm using ca. 10^−3^ M solution in DMF. Molar conductance was measured on the ELICO (CM-185) conductivity bridge using ca. 10^−3^ M solution in dry DMF by dip-type conductivity cell fitted with a platinum electrode. Magnetic susceptibility measurements were measured on a Gouy balance using Hg [Co(NCS)_4_] as the calibrant at room temperature. ESR measurement of Cu(II) complex in polycrystalline state was carried out on a JES-FA200 ESR spectrometer with X and Q band working at microwave frequency of 8.75–9.65 GHz. Thermal analysis of the complexes was carried out on a Perkin Elmer STA 6000 thermal analyzer in static air with a heating rate of 10°C/min. Powder X-ray diffraction spectrum of the complexes were recorded on Bruker AXS D8 Advance diffractometer at a wavelength 1.54 Å. All chemicals were used as received from commercial sources and solvents are purified according to the literature methods [33]. The melting points of newly synthesized compounds were determined in open glass capillary tubes and are uncorrected. Purity of the compounds was checked by TLC and the spots were observed in iodine vapor. The presence of metal and chloride contents was determined according to standard procedures [33].

Compounds, 2-thioxo-1,2-dihydroquinoline-3-carbaldehyde, and *N*-(4-phenylthiazol-2-yl)hydrazinecarboxamide were prepared according to the reported methods [31, 32].

### 2.2. Synthesis of Schiff Base Ligand ****(L)****

**Scheme 1:**
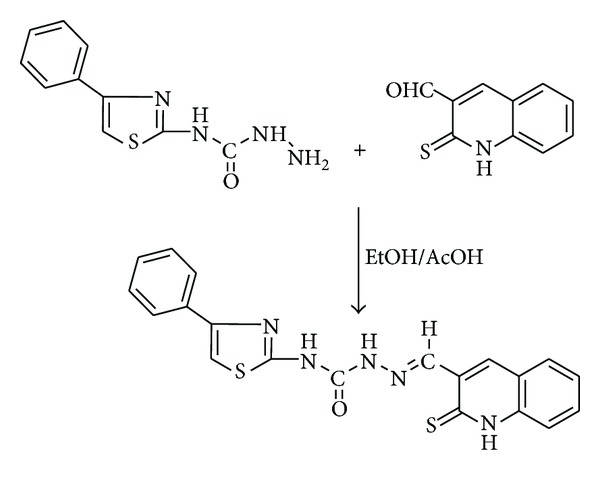
Synthesis of Schiff base ligand **(L)**.

An equimolar mixture of *N*-(4-phenylthiazol-2-yl) hydrazinecarboxamide (0.234 g, 0.001 mol) and 2-thioxo-1,2-dihydroquinoline-3-carbaldehyde (0.189 g, 0.001 mol) in ethanol (25 mL) was refluxed with a catalytic amount of glacial acetic acid (1-2 drops) for about 7-8 h on a water bath. The reaction was monitored by TLC. The reaction mixture was cooled to room temperature; pale yellow colored solid separated was filtered, washed with hot ethanol, recrystallized from 1, 4-dioxane, and dried in a desiccator over anhydrous calcium chloride at room temperature (Scheme 1). Yield: 72%; m.p. 298°C. Anal. Calc. for C_20_H_15_N_5_OS_2_ (M.W. = 405): C, 59.25; H, 3.70; N, 17.28%. Found: C, 59.18; H, 3.73; N, 17.21%. IR data (*ν* cm^−1^, KBr): *ν*(NH quinoline) 3393; *ν*(NH amide) 3259; *ν*(NH thiazole) 3119; *ν*(C=O) 1688; *ν*(C=N) 1620; *ν*(C=S) 1225. ^1^H NMR (*d*_6_-DMSO; *δ* ppm): 13.95 (s, 1H, Quinoline NH); 10.78 (s, 1H, CONH); 8.91 (s, 1H, CONH); 8.35 (s, 1H, CH=N); 7.38–8.25 (m, 11H, ArH).

### 2.3. Preparation of Cu(II), Co(II), Ni(II) and Zn(II) Complexes

Hot ethanolic solution (15 mL) of the respective metal chlorides (0.001 mol) and the Schiff base ligand (0.001 mol) in ethanol (30 mL) were refluxed for about 4-5 h on a water bath and the pH of the reaction mixture was adjusted ca. 7.0–7.5 by adding alcoholic solution of sodium acetate (0.5 g). The reaction mixture was cooled to room temperature and poured in to distilled water. Metal chelates separated were collected by filtration, washed with sufficient quantity of distilled water then with hot ethanol, and finally dried in a desiccator over anhydrous calcium chloride at room temperature.

#### 2.3.1. [Cu(C_20_H_15_N_5_OS_2_)(Cl_2_)]: Green Solid

Yield: 73%; m.p. >320°C; Anal. Calc. for [Cu(C_20_H_15_N_5_OS_2_)(Cl_2_)] (M.W. = 539.44): C, 44.49; H, 2.78; N, 12.97; Cl, 13.14; Cu, 11.77%; Found: C, 44.41; H, 2.80; N, 12.93; Cl, 13.17; Cu, 11.79%. IR data (*ν* cm^−1^, KBr): *ν*(NH quinoline) 3366; *ν*(NH amide) 3200; *ν*(NH thiazole) 3089; *ν*(C=O) 1656; *ν*(C=N) 1585; *ν*(C=S) 1188; *ν*(M–O), 514; *ν*(M–N), 454; *ν*(M–S), 372; *ν*(M–Cl), 327.

#### 2.3.2. [Co(C_20_H_15_N_5_OS_2_)(Cl_2_)]: Brown Solid

Yield: 71%; m.p. >324°C; Anal. Calc. for [Co(C_20_H_15_N_5_OS_2_)(Cl_2_)] (M.W. = 534.83): C, 44.87; H, 2.80; N, 13.08; Cl, 13.25; Co, 11.01%. Found: C, 44.95; H, 2.82; N, 13.15; Cl, 13.21; Co, 11.09%. IR data (*ν* cm^−1^, KBr): *ν*(NH quinoline) 3295; *ν*(NH amide) 3192; *ν*(NH thiazole) 3175; *ν*(C=O) 1633; *ν*(C=N) 1553; *ν*(C=S) 1215; *ν*(M–O), 536; *ν*(M–N), 448; *ν*(M–S), 356; *ν*(M–Cl), 316.

#### 2.3.3. [Ni(C_20_H_15_N_5_OS_2_)(Cl_2_)]: Brown Solid

Yield: 69%; m.p. >320°C; Anal. Calc. for [Ni(C_20_H_15_N_5_OS_2_)(Cl_2_)] (M.W. = 534.59): C, 44.89; H, 2.80; N, 13.09; Cl, 13.26; Ni, 10.97%. Found: C, 44.85; H, 2.75; N, 13.12; Cl, 13.30; Ni, 10.92%. IR data (*ν* cm^−1^, KBr): *ν*(NH quinoline) 3356; *ν*(NH amide) 3212; *ν*(NH thiazole) 3090; *ν*(C=O) 1656; *ν*(C=N) 1570; *ν*(C=S) 1216; *ν*(M–O), 522; *ν*(M–N), 476; *ν*(M–S), 337; *ν*(M–Cl), 320.

#### 2.3.4. [Zn(C_20_H_15_N_5_OS_2_)(Cl_2_)]: Orange Yellow Solid

Yield: 70%; m.p. >310°C; Anal. Calc. for [Zn(C_20_H_15_N_5_OS_2_)(Cl_2_)] (M.W. = 541.30): C, 44.33; H, 2.77; N, 12.93; Cl, 13.09; Zn, 12.08%. Found: C, 44.39; H, 2.72; N, 12.92; Cl, 13.15; Zn, 12.03%. IR data (*ν* cm^−1^, KBr): *ν*(NH quinoline) 3296; *ν*(NH amide) 3189; *ν*(NH thiazole) 3103; *ν*(C=O) 1654; *ν*(C=N) 1593; *ν*(C=S) 1216; *ν*(M–O), 540; *ν*(M–N), 485; *ν*(M–S), 359; *ν*(M–Cl), 312.^ 1^H NMR (*d*_6_-DMSO; *δ* ppm): 11.85 (s, 1H, Quinoline NH); 9.25 (s, 1H, CONH); 8.37(s, 1H, CONH); 8.19 (s, 1H, CH=N); 7.08–8.09 (m, 11H, ArH).

### 2.4. Biological Evaluation

#### 2.4.1. *In Vitro* Antimicrobial Assay

The *in vitro *antimicrobial activity of the synthesized Schiff base ligand **(L) **and its Cu(II), Co(II), Ni(II), and Zn(II) complexes were assayed against two gram negative bacterial strains *Enterobacter aerogenes *(MTCC 111) and *Pseudomonas aeruginosa *(MTCC 424) and two fungal strains *Aspergillus niger *(MTCC 282)and *Aspergillus flavus* (MTCC 277). The above organisms were obtained from the Department of Microbiology and Biotechnology, Gulbarga University, Gulbarga, Karnataka, India, which are previously procured from Institute of Microbial Technology Chandigarh, India. The stock solutions were prepared by dissolving 10 mg of each test compound in 10 mL of freshly distilled DMSO. The various concentrations of the test compounds (100, 50 and 25 *μ*g mL^−1^) were prepared by diluting the stock solution with the required volume of freshly distilled DMSO. The diameter of the zone of inhibition generated by each of the test compounds against bacterial and fungal growth was measured using antibiogram zone measuring scale.

*(1) Agar Well Diffusion Assay*. *In vitro *antibacterial and antifungal activities of synthesized Schiff base ligand **(L) **and its Cu(II), Co(II), Ni(II), and Zn(II) complexes were determined by standard agar well diffusion assay. Mueller-Hinton agar media were used for antibacterial studies. The pure dehydrated Mueller-Hilton agar (38 g) dissolved in 1000 mL of distilled water. The pure cultures of the bacterial strains* Enterobacter aerogenes *and *Pseudomonas aeruginosa *were subcultured by inoculating in the nutrient broth and were incubated at 37°C for about 18 h. The agar plates were prepared by using the above media and wells were dug with the help of 6 mm sterile metallic cork borer. Each plate was inoculated with 18 h old bacterial culture (100 *μ*L) using a micropipette and spread uniformly using bent glass rod on each plate. Different concentrations of the test compounds (100, 50, and 25 *μ*g mL^−1^) were incorporated into the wells using micropipette and the plates were kept for incubation at 37°C for 24 h. After the completion of incubation period, the diameters of the inhibition zones generated by each test compound against bacterial growth were measured using antibiogram zone measuring scale. The experiment done in triplicate and the average values were calculated for antibacterial activity.

Potato dextrose agar (PDA) media were used for the antifungal studies. The following ingredients were used to prepare the medium. Potatoes (sliced, washed, unpeeled 200 g), dextrose (20 g), agar (20 g) in 1000 mL distilled water. The pure cultures *Aspergillus niger *and *Aspergillus flavus *were inoculated on PDA slants. These slants were incubated at 32°C for 7 days. To these 7-day-old slants of fungal strains, 10 mL of 0.1% tween-80 solution was added and the culture was scraped with sterile inoculating loop to get uniform spore suspension. The agar plates were prepared by using the above potato dextrose agar media and wells were dug with the help of 6 mm sterile metallic cork borer. Each plate was inoculated with 7-day-old spore suspension of each fungal culture (100 *μ*L) using a micropipette and spread uniformly using bent glass rod on each plates. Each well was incorporated with the test compound solution of different concentrations (100, 50, and 25 *μ*g mL^−1^). All the inoculated plates were incubated at 32°C for about 48 h. After the completion of incubation period, the diameters of the inhibition zones generated by each test compound against fungal growth were measured using antibiogram zone measuring scale. The experiment done in triplicate and the average values were calculated for antifungal activity.

*(2) Minimum Inhibitory Concentration (MIC)*. Minimum inhibitory concentration (MIC) was defined as the lowest concentration where no visible turbidity was observed in the test tubes [34, 35]. Minimum inhibitory concentration of the compounds was determined in nutrient agar plate by microdilution method according to the National Committee for Clinical Laboratory Standards [36]. Standardized suspension of test organisms (0.1 mL, 10^6^ cfu/mL) and synthesized Schiff base ligand **(L) **and its Cu(II), Co(II), Ni(II), and Zn(II) complexes in different concentrations (100, 50, 25, 12.50, 6.25, 3.125, 1.563, 0.78, 0.39, and 0.195 *μ*g/mL) were taken in test tubes and test tubes with Gentamycin and Fluconazole as positive control for bacterial and fungal strains, respectively. DMSO is used as a negative control for antibacterial and antifungal, respectively. The bacterial tubes were incubated at 37°C for 18 h and fungal tubes were incubated at 32°C for 48 h. The lowest concentration that produced no visible bacterial growth compared with the control tubes was regarded as MIC.

#### 2.4.2. DNA Cleavage Activity

In order to study whether newly synthesized Schiff base ligand and its metal complexes could behave as DNA cleaving agents or not, they were examined using plasmid pBR322 DNA (Bangal re Genei, Bengaluru, Cat. No 105850) as a target molecule according to the literature method [37].

The cleavage activity of the test compounds was analyzed by agarose gel electrophoresis method. The 600 mg of agarose was dissolved in 60 mL of TAE buffer (4.84 g Tris base, pH 8.0, 0.5 M EDTA) by boiling. When the gel attains approximately 55°C, it was poured into the gel cassette fitted with comb. The gel was allowed to solidify and then carefully the comb was removed. The gel was placed in the electrophoresis chamber flooded with TAE buffer. Test compounds were prepared in DMSO (1 mg mL^−1^). The test compounds were added separately to the isolated plasmid pBR322 DNA (225 ng) and incubated for 2 h at 37°C. After the incubation period, the 20 *μ*L of DNA sample (mixed with bromophenol blue dye at a 1 : 1 ratio) was loaded carefully into the electrophoresis chamber wells along with standard DNA marker and a constant electricity of 50 V passed for about 30 min. The gel was removed carefully and stained with Ethidium bromide (EtBr) solution (10 *μ*g/mL) for 10–15 min. The bands were observed under UV transilluminator (UVP, Germany) and photographed to determine the extent of DNA cleavage, and the results were compared with those of a standard DNA marker.

#### 2.4.3. *In Vitro* Cytotoxicity

The brine shrimp lethality bioassay has been chosen to evaluate the *in vitro* cytotoxic effect of the newly synthesized Schiff base ligand **(L)** and its Cu(II), Co(II), Ni(II), and Zn(II) complexes by using the protocol of Meyer et al. [38]. This is an efficient, rapid, inexpensive test and has a good correlation with cytotoxic activity.

Brine shrimp (*Artemia salina*) eggs were hatched in a shallow rectangular plastic dish (22 × 32 cm) filled with artificial seawater, which was prepared with a commercial salt mixture and double distilled water. An unequal partition was made in the plastic dish with the help of a perforated device. Approximately 50 mg of eggs were sprinkled into the large compartment, which was darkened while the minor compartment was open to ordinary light. After two days nauplii were collected by a pipette from the lighted side. A sample of the test compound was prepared by dissolving 20 mg of each compound in 2 mL of DMSO. From this stock solution 100, 50, and 25 *μ*g mL^−1^ were transferred to nine vials (three for each dilution were used for each test sample and LD_50_ is the mean of three values) and one vial was kept as control having 2 mL of DMSO only. The solvent was allowed to evaporate overnight. After two days, when shrimp larvae were ready, 1 mL of seawater and 10 shrimps were added to each vial (30 shrimps/dilution) and the volume was adjusted with seawater to 10 mL per vial. After 24 h the number of survivors was counted. Data were analysed by a Finney computer program to determine the LD_50_ values [39].

## 3. Results and Discussion

The reaction of Schiff base ligand **(L)** with Cu(II), Co(II), Ni(II), and Zn(II) ions in 1 : 1 ratio resulted in the complex of the type [ML(Cl)_2_]. The physical and analytical data agree well with the proposed composition of Schiff base ligand and its Cu(II), Co(II), Ni(II), and Zn(II) complexes. The newly synthesized complexes are colored solids, stable in air, and insoluble in water and common organic solvents but completely soluble in DMF and DMSO. The molar conductance data of the complexes was measured in DMF at ca. 10^−3^ M and all the complexes showed conductance in the range of 50–61 Ohm^−1^ cm^2^ mol^−1^ at room temperature indicating nonelectrolytic nature of the complexes suggesting that the Cl^−^ anion is coordinated to metal ion. This was further supported by the proposed general formula of the complexes based upon the results of elemental analysis (Table 1) and spectral data.

### 3.1. IR Spectra

IR spectrum of the ligand showed a high intensity band at 1688 cm^−1^ due to *ν*(C=O) and three absorption bands at 3393, 3259, and 3119 cm^−1^ due to quinoline NH, amide NH, and NH attached to thiazole moiety, respectively. A high intensity band observed at 1620 cm^−1^ is attributed to the azomethine *ν*(C=N) vibration and a band at 1225 cm^−1^ to *ν*(C=S) functioning at 2-position of quinoline moiety.

In order to study the binding mode of the Schiff base to the metal ion in complexes, the IR spectrum of the free ligand was compared with the spectra of the complexes. In the IR spectra of the complexes, medium intensity weak bands at 3366–3295, 3212–3189, and 3175–3089 cm^−1^ were due to quinoline NH, amide NH, and NH attached to thiazole moiety, respectively, which appeared at about the same region as in the case of ligand indicating their noninvolvement in coordination. The shift of amide carbonyl *ν*(C=O) to lower frequency side about 55–32 cm^−1^ which appeared in the region 1656–1633 cm^−1^ in all the complexes confirms the coordination of oxygen atom of amide C=O with the metal ions as such without undergoing enolization [40]. The IR spectrum of all the complexes showed a shift of the azomethine *ν*(C=N) band towards lower frequency side about 67–27 cm^−1^ and appeared in the region 1593–1553 cm^−1^ when compared with the free ligand indicating the coordination of the azomethine nitrogen to the metal ions [41]. The shift of band due to *ν*(C=S) in all the complexes towards lower frequency side by 37–9 cm^−1^ when compared to the ligand, which appeared in the region 1215–1188 cm^−1^, proves the coordination of the sulfur atom of quinoline 2-thione to metal ions. The formation of complex was further confirmed by the appearance of new bands in the regions 540–514, 485–448, 372–337, and 327–312 cm^−1^ in all the complexes due to skeletal metal-oxygen, metal-nitrogen, metal-sulfur, and metal-chloride vibrations, respectively. The important IR spectral data of the Schiff base ligand and its metal complexes are represented in Table 2.

### 3.2. ^1^H NMR Spectra

The ^1^H NMR spectrum of ligand displayed four distinct singlets at *δ* 13.95 (s, 1H, quinoline NH), *δ* 10.78 (s, 1H, CONH), *δ* 8.91 (s, 1H, CONH), and *δ* 8.35 (s, 1H, CH=N) and eleven aromatic protons as multiplets in the region *δ* 7.38–8.25 (m, 11H, ArH).

The ^1^H NMR of Zn(II) complex displayed four distinct singlets at *δ* 11.85 (s, 1H, quinoline NH), *δ* 9.25 (s, 1H, CONH), 8.37 (s, 1H, CONH), and 8.19 (s, 1H, CH=N) and eleven aromatic protons as multiplets in the region 7.08–8.09 (m, 11H, ArH). The ^1^H NMR spectral data of Schiff base ligand and its Zn(II) complex confirm the formation of Zn(II) complex with the ligand.

### 3.3. ESI Mass Spectral Data

**Scheme 2:**
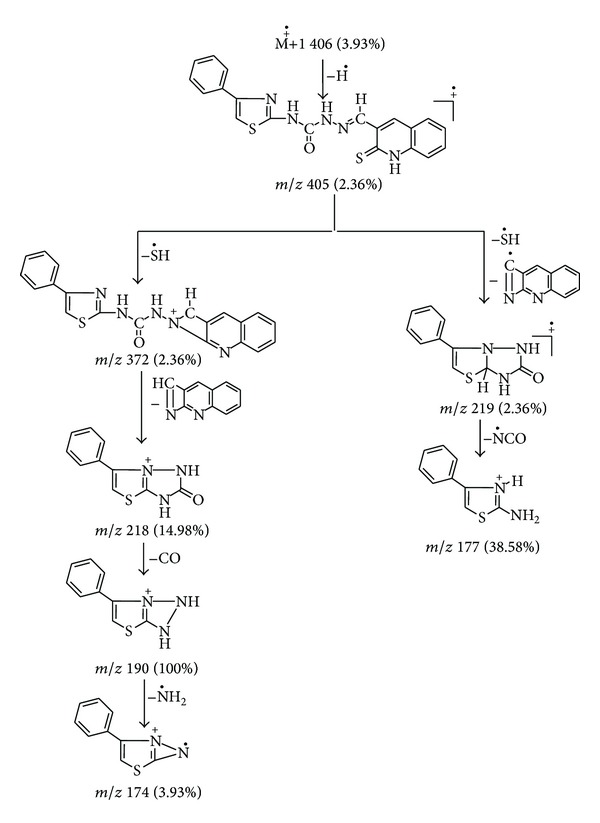
Mass fragmentation pattern of Schiff base ligand **(L)**.

**Figure 1:**
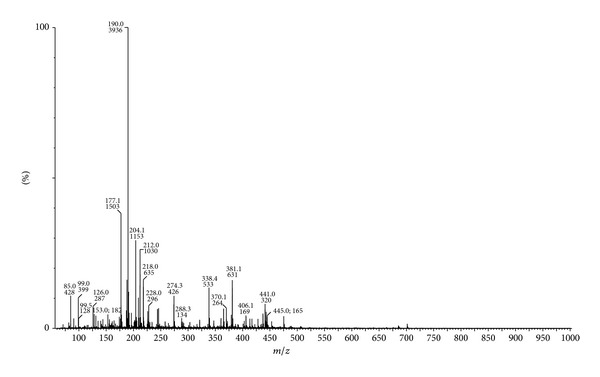
ESI mass spectrum of Schiff base ligand **(L)**.

The ESI mass spectra of the Schiff base ligand **(L)** and its Co(II) and Ni(II) complexes are performed to determine their molecular weight and study their fragmentation pattern. The mass spectrum of ligand showed a peak recorded at *m*/*z* 406 (3.93%) due to M^+∙^ + 1 (Figure 1). This on loss of hydrogen radical gave a peak at *m*/*z* 405 (2.36%) which is equivalent to its molecular weight (M.W. = 405). Further, this molecular ion underwent fragmentation by two routes. First, on loss of SH radical, it gave a fragment ion peak recorded at *m*/*z* 372 (2.36%), followed by expulsion of C_10_H_6_N_2_ molecule which gave a fragment ion peak recorded at *m*/*z* 218 (14.98%). This fragment ion on further expulsion of CO molecule gave a fragment ion peak at *m*/*z* 190 (100%) which is also a base peak. This base peak on loss of NH_2_ radical gives a fragment ion peak recorded at *m*/*z* 174 (3.93%). The molecular ion underwent fragmentation in another route wherein it losses SH radical and C_10_H_5_N_2_ radical simultaneously giving a fragment ion peak recorded at *m*/*z* 219 (2.36%). This fragment ion on further loss of NCO radical gave a fragment ion peak recorded at *m*/*z* 177 (38.58%). This schematic mass spectral fragmentation pattern of ligand is in consistency with its structure which is depicted in Scheme 2.

**Scheme 3:**
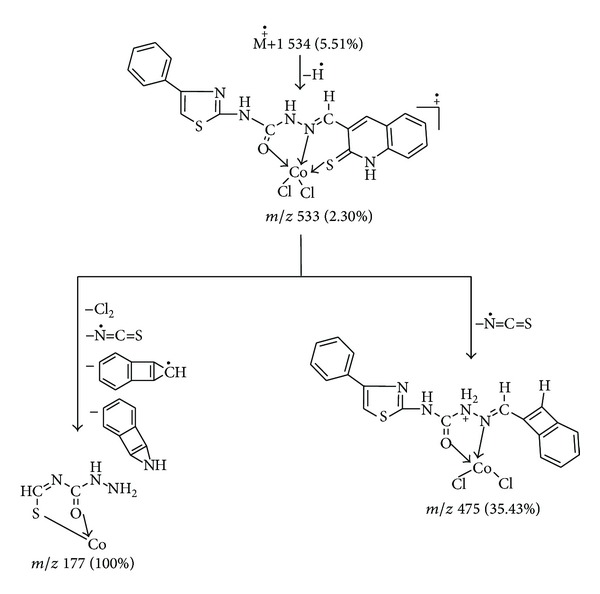
Mass fragmentation pattern of Co(II) complex.

The ESI mass spectrum of Co(II) complex exhibited a peak due to M^+∙^ + 1 at *m*/*z* 534 (5.51%). This on loss of hydrogen radical gave a peak at *m*/*z* 533 (2.30%), which is equivalent to its molecular weight (M.W. = 533). This molecular ion underwent fragmentation by two routes. First, on simultaneous loss of chlorine molecule, N=C=S radical, C_9_H_5_ radical, and C_8_H_5_N species gave a fragment ion peak recorded at *m*/*z* 177 (100%) which is also a base peak. In another route, the molecular ion peak on loss of N=C=S radical gave a fragment ion peak recorded at *m*/*z* 475 (35.43%). The schematic mass spectral fragmentation pattern of Co(II) complex of ligand is in consistency with its structure which is depicted in Scheme 3.

**Scheme 4:**
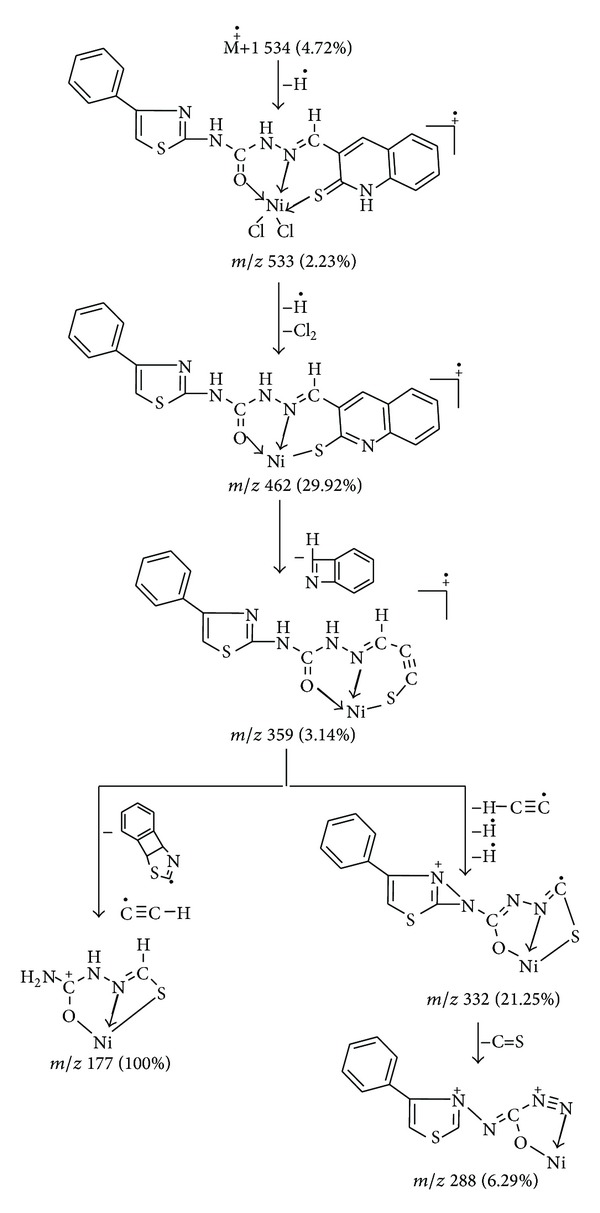
Mass fragmentation pattern of Ni(II) complex.

**Figure 2:**
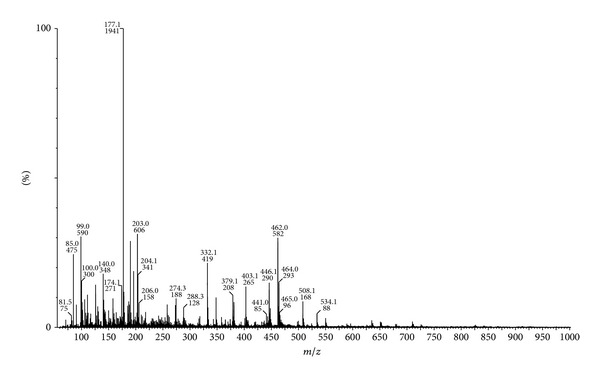
ESI mass spectrum of Ni(II) complex.

Similarly, the mass spectra of Ni(II) complex exhibited a peak at *m*/*z* 534 (4.72%) due to M^+∙^ + 1 (Figure 2). This is on loss of hydrogen radical gave the fragment ion peak recorded at *m*/*z* 533 (2.23%), which is equivalent to its molecular weight (M.W. = 533). Further this molecular ion underwent fragmentation by the loss of hydrogen radical and chlorine molecule simultaneously giving a fragment ion peak recorded at *m*/*z* 462 (29.92%). This on further loss of C_7_H_5_N molecule gave a fragment ion peak recorded at *m*/*z* 359 (3.14%), which underwent fragmentation by two routes. First, on loss of H–C*≡*C radical and two hydrogen radicals it simultaneously gave a fragment ion peak recorded at *m*/*z* 332 (21.25%), which on further expulsion of C=S molecule gave a fragment ion peak recorded at *m*/*z* 288 (6.29%). In another route, fragment ion peak recorded at *m*/*z* 359 (3.14%) on simultaneous loss of C_9_H_4_NS radical and C*≡*C–H radical gave a fragment ion peak recorded at *m*/*z* 177 (100%) which is also a base peak. The schematic mass spectral fragmentation pattern of Ni(II) is in consistency with its structure which is depicted in Scheme 4.

### 3.4. Electronic Spectra and Magnetic Susceptibility

The electronic spectra of the Cu(II), Co(II), and Ni(II) complexes were recorded in DMF solution at ca. 10^−3^ M at room temperature. The band positions of absorption band maxima assignments are listed in Table 3. The electronic spectra of green colored Cu(II) complex showed three absorption bands around 9993 cm^−1^, 14595 cm^−1^, and 18045 cm^−1^ which are assigned to ^2^B_1_ → ^2^A_1_ (*ν*_1_), ^2^B_1_ → ^2^B_2_ (*ν*_2_), and ^2^B_1_ → ^2^E (*ν*_3_) transitions, respectively. The observed transitions for Cu(II) complex are well within the ranges of 9000–10000 cm^−1^ (*ν*_1_), 11500–16000 cm^−1^ (*ν*_2_), and 15000–19000 cm^−1^ (*ν*_3_) for Cu(II) complexes of square pyramidal geometry [42, 43]. The electronic spectra of brown colored Co(II) complex displayed three absorption bands at 11098 cm^−1^, 17675 cm^−1^, and 20180 cm^−1^ which are assigned to  ^4^A_2_ + ^4^E → ^4^B_1_ (*ν*_1_), ^4^A_2_ + ^4^E → ^4^E(P) (*ν*_2_), and  ^4^A_2_ + ^4^E → ^4^A_2_(P) (*ν*_3_) transitions, respectively, suggesting the square pyramidal geometry of the Co(II) complex [44]. The brown colored Ni(II) complex under present investigation displayed three absorption band at 10000 cm^−1^, 13543 cm^−1^, and 22307 cm^−1^ which are assigned to transitions ^3^B_1_ → ^3^E^a^ (*ν*_1_), ^2^B_1_ → ^3^A_2 _(*ν*_2_), and ^3^B_1_ → ^3^E^b^ (*ν*_3_), respectively, indicating the square pyramidal geometry of the Ni(II) complex [45].

The magnetic moment value for Cu(II) complex was found to be 1.73 BM which is in the range of 1.71–1.76 BM, agreeing well with the spin value of S = 1/2, as usually observed for Cu(II) complex, which supports its square pyramidal geometry [46]. The magnetic moment value for Co(II) complex was found to be 4.31 BM which is in the range 4.3–4.6 BM, for five coordinate square pyramidal geometries of Co(II) complex [47]. The magnetic moment of Ni(II) complex was found to be 2.78 BM, which is well within the range known for five coordinated square pyramidal geometries of Ni(II) complex [45].

### 3.5. ESR Spectra

**Figure 3:**
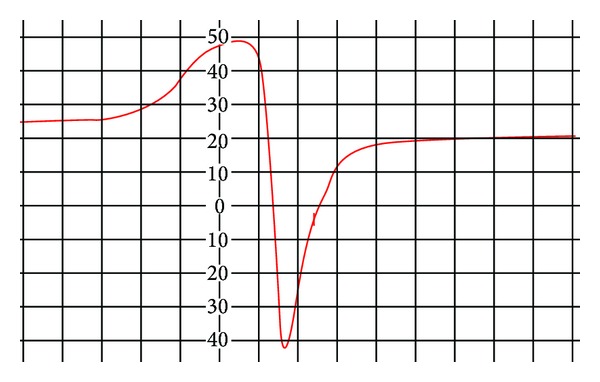
ESR spectrum of Cu(II) complex.

In order to obtain further information about the stereochemistry and site of the metal-ligand bonding and determine the magnetic interaction in the metal complexes, the X-band ESR spectrum of Cu(II) complex has been recorded in the polycrystalline state at room temperature using DPPH as a standard. The ESR spectrum of Cu(II) complex exhibited a single broad signal (Figure 3) due to dipolar broadening and enhanced spin lattice relaxation. The ESR spectra of Cu(II) complex exhibited auxiliary symmetric *g*-tensor parameter with *g*_||_(2.16) > *g*_⊥_(2.03) > 2.0023, indicating presence of unpaired electron in *d*_*x*2−*y*2_ ground state characteristic of square pyramidal geometry. The averaged “*g*” value for overall distortion is calculated using the equation: *g*_avg_ = (1/3)(2*g*_⊥_ + *g*_||_). The exchange interaction parameter *G* is calculated using the equation: *G* = *g*_||_ − 2.0023/*g*_⊥_ − 2.0023. According to Hathaway and Billing [48], if the value of *G* is more than four, the exchange interaction between the Cu centres is negligible. In the present case the *G* value of 5.01 confirms the that exchange interactions between Cu(II) centers in solid state are negligible [49].

### 3.6. Thermal Studies

In order to examine the thermal stability of the complexes, thermo gravimetric (TG) and differential thermal analyses (DTA) were carried out for Cu(II), Co(II), and Zn(II) complexes in static air at a temperature range between 40 to 750°C at the heating rate of 10°C min^−1^. The proposed stepwise thermal degradation pattern of complexes with temperature and formation of metal oxides is given in Table 4.

#### 3.6.1. Cu(II) Complex

**Figure 4:**
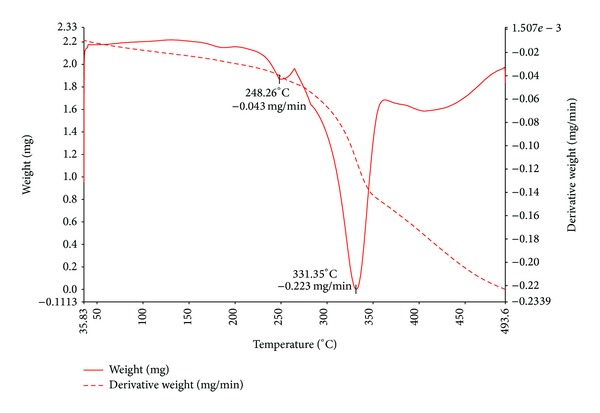
TGA and DTA curve of Cu(II) complex.

TGA and DTA curves of Cu(II) complex (Figure 4) showed that the complex is stable up to 237°C and no weight loss is observed before this temperature. The first stage of degradation occurred at 237.6°C, with the loss of the two chlorine atoms with a practical weight loss of 11.96% (Calc.12.99%). The resultant complex on further degradation gave a break at 291°C by the loss of NCS species of quinoline moiety with a practical weight loss of 11.41% (Calc. 12.37%). The resultant complex underwent third stage of decomposition at 341°C due to loss of C_9_H_7_ molecule of quinoline and C_6_H_6_ molecule of thiazole moiety simultaneously with a practical weight loss of 47.96% (Calc. 47.01%). Further, complex showed decomposition up to 493°C due to the loss of remaining organic moiety. The final weight of the residue corresponds to cupric oxide.

#### 3.6.2. Co(II) Complex

The thermogram of Co(II) complex showed first stage of decomposition due to loss of NCS species of quinoline moiety at 258°C with a practical weight loss of 9.66% (Calc. 10.86%). The resultant complex on further degradation gave a break at 320°C by the loss of C_8_H_6_ molecule of quinoline moiety with a practical weight loss of 22.22% (Calc. 21.43%), which on further degradation gave a break at 458°C due to loss of remaining thiazole moiety (C_9_H_7_N_2_S), two chlorine atoms, and HC=N–N–NH group simultaneously with a practical weight loss of 78.02% (Calc. 76.75%). After this, the complex showed a gradual decomposition up to 697°C by the loss of remaining organic moiety. The final weight of the residue corresponds to cobalt oxide.

#### 3.6.3. Zn(II) Complex

The thermogram of Zn(II) complex showed first stage of decomposition due to loss of C_6_H_7_ species of thiazole and two chlorine atoms at 270°C with a practical weight loss of 28.71% (Calc. 27.57%). This on further loss due to C_10_H_6_NS of quinoline and CH=CH molecule of thiazole moiety gave a break at 320°C with a practical weight loss of 51.58% (Calc. 50.58%). After this, the complex showed a gradual decomposition up to 351°C by the loss of remaining organic moiety. The final weight of the residue corresponds to respective metal oxide.

### 3.7. Powder X-Ray Diffraction Studies

The synthesized Cu(II), Co(II), Ni(II), and Zn(II) complexes of Schiff base ligand **(L)** were soluble in some polar organic solvents (DMSO and DMF). The crystals that are suitable for single crystal studies are not obtained. In order to test the degree of crystallinity of the synthesized metal complexes, we obtained the powder X-ray diffraction pattern of the above complexes. The X-ray diffraction of Cu(II), Co(II), Ni(II), and Zn(II) complexes was scanned in the range 3–80°(*θ*) at wave length 1.54 Å. In all the complexes, the trend of the curves decreases from maximum to minimum intensity indicating the amorphous nature of the complexes in the present metal-ligand formation.

**Figure 5:**
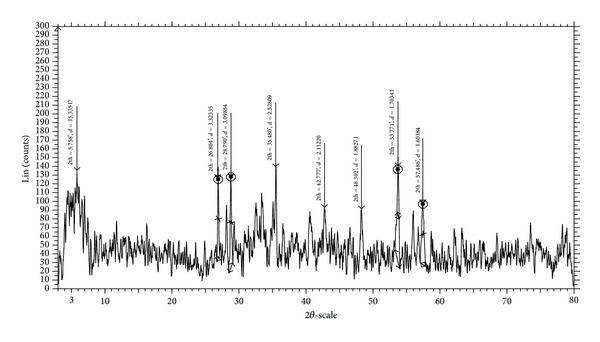
Powder XRD spectrum of Cu(II) complex.

The X-ray diffraction pattern of Cu(II) complex records eight reflections between the range 3–80°(2*θ*), which arise from diffraction of X-ray by the plane of the complex (Figure 5). The interplanar spacing (*d*-values) has been calculated by using Bragg's equation: *nλ* = 2*d* sin*θ*. The unit cell calculations have been done for cubic symmetry from the all-important peaks and the methods yielded *h* *k* *l* (Miller indices) unit cell parameter values and depicted in Table 5. The observed interplanar *d*-spacing values have been compared with the calculated ones and found to be in good agreement. The *h* ^2^ + *k* ^2^ + *l* ^2^ values are 1, 21, 25, 37, 53, 66, 81, and 92. It was observed that the presence of forbidden number 92 indicates that the Cu(II) complex may belong to hexagonal or tetragonal system.

**Figure 6:**
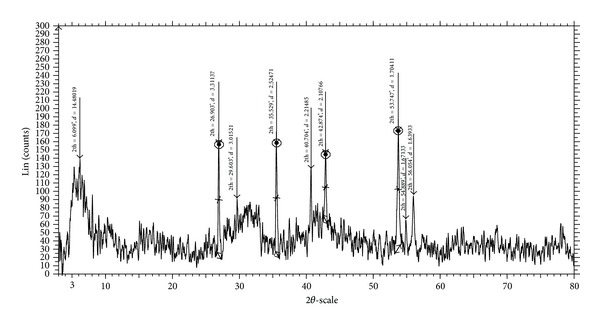
Powder XRD spectrum of Ni(II) complex.

The X-ray diffraction pattern of Ni(II) complex records nine reflections between the range 3–80°(2*θ*), which arise from diffraction of X-ray by the plane of the complex (Figure 6). The interplanar spacing (*d*-values) has been calculated by using Bragg's equation: *nλ* = 2*d* sin*θ*. The unit cell calculations have been done for cubic symmetry from the all-important peaks and the methods yielded *h* *k* *l* (Miller indices) unit cell parameter values depicted in Table 6. The observed interplanar *d*-spacing values have been compared with the calculated ones and found to be in good agreement. The *h* ^2^ + *k* ^2^ + *l* ^2^ values are 1, 19, 23, 33, 43, 47, 72, 75, and 78. It was observed that the presence of forbidden numbers 23, 43, and 47 indicates that the Ni(II) complex may belong to hexagonal or tetragonal system.

Similar calculations were done for Co(II) and Zn(II) complexes. The Co(II) complex showed seven reflections and Zn(II) complex showed five reflections between the range 3–80°(2*θ*). For all the complexes, the interplanar spacing (*d*-values) and unit cell calculations have been done for cubic symmetry from the all-important peaks and the method yielded *h* *k* *l* (Miller indices) units cell parameter values. The *h* ^2^ + *k* ^2^ + *l* ^2^ values for Co(II) complex are 1, 5, 7, and 10 and for the Zn(II) complex 1, 2, 29, 37, and 63, respectively. It was observed that the presence of forbidden number 7 in Co(II) complex and forbidden number 63 in Zn(II) complex, respectively, indicates that these complexes may belong to hexagonal or tetragonal system.

### 3.8. Biological Evaluation

#### 3.8.1. *In Vitro* Antibacterial and Antifungal Activities

The *in vitro* antimicrobial activity of all the synthesized compounds was screened against *Enterobacter aerogenes *and *Pseudomonas aeruginosa* bacteria and *Aspergillus niger* and *Aspergillus flavus *fungal strains by minimum inhibitory concentration (MIC) method. The minimum inhibitory concentration (MIC) profiles of all the compounds against bacteria and fungi are summarized in Table 7.

The MIC values indicated that all the complexes exhibited promising results compared to the ligand against mentioned microorganisms, and this activity is found to be enhanced on coordination with the metal ions. This enhancement in the activity may be rationalized on the basis that ligands mainly possess C=N bond. The enhanced activity of the complexes over the ligand can be explained on the basis of chelation theory [50, 51]. It is observed that, in a complex, the positive charge of the metal is partially shared with the donor atoms present in the ligand, and there may be *π*-electron delocalization over the whole chelate [52]. This increases the lipophilic character of the metal chelate and favors its permeation through the lipoid layer of the bacterial membranes. The heterocyclic Schiff bases with different functional groups have greater tendency to interact with nucleoside bases even after complexation with metal ion or with the essential metal ions present in the biosystem can act as a promising bactericides because they always tend to interact with enzymatic functional groups in order to achieve higher coordination numbers [53]. There are also other factors which increase the activity, namely, solubility, conductivity, and bond length between the metal and the ligand.

#### 3.8.2. DNA Cleavage Activity

**Figure 7:**
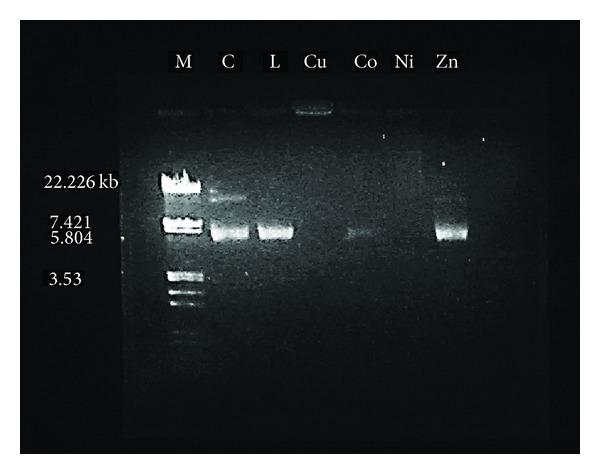
DNA cleavage on plasmid pBR 322: M: Standard DNA, C: Control DNA (untreated pBR 322), L: Schiff base ligand, Cu: Cu(II) complex, Co: Co(II) complex, Ni: Ni(II) complex, and Zn: Zn(II) complex.

The interaction of plasmid pBR322 DNA with newly synthesized ligand **L** and its Cu(II), Co(II), Ni(II), and Zn(II) complexes was studied using agarose gel electrophoresis method. The gel picture showing the cleavage of plasmid pBR322 DNA is depicted in Figure 7. The characterization of DNA recognition by transition metal complex has been aided by the DNA cleavage chemistry associated with redox-active or photo activated metal complexes [54]. The electrophoresis analysis clearly revealed that the ligand and its metal complexes have acted on DNA because of a difference in molecular weight between the control and the treated DNA samples. The difference was observed in the bands of lanes of complexes compared with the control DNA of pBR322 due to the relaxation of circular DNA into linear form. This shows that the control DNA alone does not show any apparent cleavage, whereas the ligand and its metal complexes do show. In the present study, the Ethedium bromide (EtBr) stained banding pattern of plasmid pBR322 DNA was tested with newly synthesized ligand and its metal complexes. In the present case, the ligand and its Cu(II), Co(II), and Zn(II) complex showed complete cleavage of super coiled DNA and the Ni(II) complex showed partial cleavage of relaxed DNA and complete cleavage of supercoiled DNA. This clearly reveals the important role of coordination of O, N, and S groups to the metal ion in these DNA cleavage activities. On the basis of these findings, it can be concluded that all the newly synthetized compounds under present study are good pathogenic microorganism inhibitor; as evident on the DNA cleavage of pBR322.

#### 3.8.3. *In Vitro* Cytotoxicity

All the synthesized compounds were screened for their cytotoxicity (brine shrimp bioassay) using the protocol of Meyer et al. [38]. From the data recorded in Table 8, it is evident that all the newly synthesized metal complexes exhibited potent activity when compared to the free ligand. The Co(II) and Ni(II) complexes displayed significant potent cytotoxic activity as LD_50_ = 1.168 × 10^−4^ and 1.074 × 10^−4^ M/mL, respectively, against *Artemia salina*.

## 4. Conclusions

**Figure 8:**
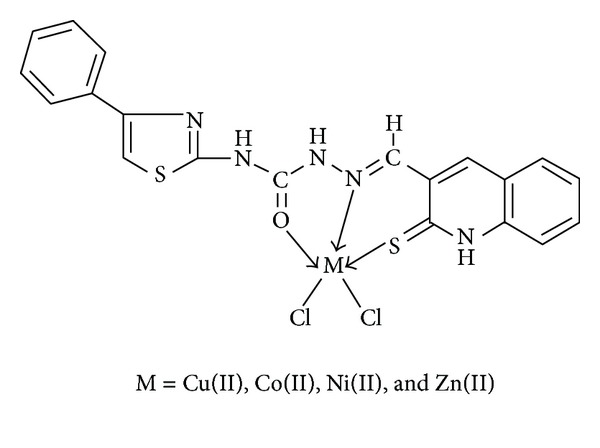
Proposed structure of the complexes.

The newly synthesized Schiff base ligand *N*-(4-phenylthiazol-2yl)-2-((2-thiaxo-1,2-dihydroquinolin-3-yl)methylene)hydrazinecarboxamide behaves as tridentate ONS donor and forms the complexes of type [ML(Cl)_2_]. With the help of various physicochemical and spectroscopic methods such as IR, ^1^H NMR, UV-Visible, and ESR, the square pyramidal geometries of the Cu(II), Co(II), Ni(II), and Zn(II) complexes have been proposed (Figure 8). The newly synthesized metal complexes showed good antimicrobial activity when compared to the free ligand. The DNA cleavage activity of all the synthesized compounds showed the cleavage of plasmid DNA pBR 322 and the cytotoxicities of Co(II) and Ni(II) complexes indicate potent cytotoxic agents that might become potent anticancer agent in clinical trials.

## Figures and Tables

**Table 1:** Physical, Analytical and Molar conductance data of Schiff base ligand **(L)** and its metal complexes.

Compound	M.W.^a^	M.P. (°C)^b^	Elemental Analysis, found (Calc.) [%]					*λ*_*m*_ (cm^2^ *Ω* ^−1^ mol^−1^)	*μ*_eff_ (BM)	Color
			M	C	H	N	Cl			
C_20_H_15_N_5_OS_2_	405	298	—	59.18 (59.25)	3.73 (3.70)	17.21 (17.28)	—	—	—	Yellow
[Cu(C_20_H_15_N_5_OS_2_)(Cl_2_)]	539.44	>320	11.79 (11.77)	44.41 (44.49)	2.80 (2.78)	12.93 (12.97)	13.17 (13.14)	50	1.73	Green
[Co(C_20_H_15_N_5_OS_2_)(Cl_2_)]	534.83	>324	11.09 (11.01)	44.95 (44.87)	2.82 (2.80)	13.15 (13.08)	13.21 (13.25)	61	4.31	Brown
[Ni(C_20_H_15_N_5_OS_2_)(Cl_2_)]	534.59	>320	10.92 (10.97)	44.85 (44.89)	2.75 (2.80)	13.12 (13.09)	13.30 (13.26)	56	2.78	Brown
[Zn(C_20_H_15_N_5_OS_2_)(Cl_2_)]	541.30	>310	12.03 (12.08)	44.39 (44.33)	2.72 (2.77)	12.92 (12.93)	13.15 (13.09)	55	Dia.^c^	Orange Yellow

^a^Molecular weight of the compounds.

^b^Melting point of the compounds at their decomposition.

^c^Diamagnetic.

**Table 2:** IR spectral data of Schiff base ligand **(L) **and its metal complexes.

Compounds	Quinoline *ν*_(NH)_ cm^−1^	Amide *ν*_(NH)_ cm^−1^	Thiazole *ν*_(NH)_ cm^−1^	*ν*_(C=O)_ cm^−1^	*ν*_(C=N)_ cm^−1^	*ν*_(C=S)_ cm^−1^	*ν*_(M–O)_ cm^−1^	*ν*_(M–N)_ cm^−1^	*ν*_(M–S)_ cm^−1^	*ν*_(M–Cl)_ cm^−1^
C_20_H_15_N_5_OS_2_	3393	3259	3119	1688	1620	1225	—	—	—	—
[Cu(C_20_H_15_N_5_OS_2_)(Cl_2_)]	3366	3200	3089	1656	1585	1188	514	454	372	327
[Co(C_20_H_15_N_5_OS_2_)(Cl_2_)]	3295	3192	3175	1633	1553	1215	536	448	356	316
[Ni(C_20_H_15_N_5_OS_2_)(Cl_2_)]	3356	3212	3090	1656	1570	1216	522	476	337	320
[Zn(C_20_H_15_N_5_OS_2_)(Cl_2_)]	3296	3189	3103	1654	1593	1216	540	485	359	312

**Table 3:** Electronic and ESR spectral data.

Complexes	Electronic spectra		ESR spectral data			
	Absorption (cm^−1^)	Band assignment	*g*_⊥_	*g*_||_	*g*_avg_	*G*
[Cu(C_20_H_15_N_5_OS_2_)(Cl_2_)]	9993	^ 2^B_1_→^2^A_1_ (*ν*_1_)	2.03	2.16	2.07	5.01
	14595	^ 2^B_1_→^2^B_2_ (*ν*_2_)
	18045	^ 2^B_1_→^2^E (*ν*_3_)

[Co(C_20_H_15_N_5_OS_2_)(Cl_2_)]	11098	^ 4^A_2_ + ^4^E→^4^B_1_ (*ν*_1_)
	17675	^ 4^A_2_ + ^4^E→^4^E (P) (*ν*_2_)
	20180	^ 4^A_2_ + ^4^E→^4^A_2_ (P) (*ν*_3_)

[Ni(C_20_H_15_N_5_OS_2_)(Cl_2_)]	10000	^ 3^B_1_→^3^E^a^ (*ν*_1_)
	13543	^ 2^B_1_→^3^A_2_ (*ν*_2_)
	22307	^ 3^B_1_→^3^E^b^ (*ν*_3_)

**Table 4:** Thermal data of Cu(II), Co(II) and Zn(II) complex.

Complex	Decomposition temp. (°C)	Weight loss (%)		Metal oxide (%)		Inference
		Obsd.	Calc.	Obsd.	Calc.	
[Cu(C_20_H_15_N_5_OS_2_)(Cl_2_)]	237.6	11.96	12.99	—	—	Loss due to two chlorine atoms
	291	11.41	12.37	—	—	Loss due to NCS species
	341	47.96	47.01	—	—	Loss due to C_9_H_7_ molecule of quinoline and C_6_H_6_ molecule of thiazole moiety
	Up to 493	—	—	15.92	15.65	Loss due to remaining organic moiety

[Co(C_20_H_15_N_5_OS_2_)(Cl_2_)]	258	9.66	10.86	—	—	Loss due to NCS species
	320	22.22	21.43	—	—	Loss due to C_8_H_6_ molecule of quinoline moiety
	458	78.02	76.75	—	—	Loss due to C_9_H_7_N_2_S of thiazole moiety, two chlorine atoms and HC=N–NH group
	Up to 697			17.15	17.56	Loss due to remaining organic moiety

[Zn(C_20_H_15_N_5_OS_2_)(Cl_2_)]	270	28.71	27.57	—	—	Loss due C_ 6 _H_ 7 _ species of thiazole and two chlorine atoms
	320	51.58	50.58	—	—	Loss due to C_ 10 _H_ 6 _NS molecule of quinoline and CH=CH molecule of thiazole moiety
	Up to 351	—	—	16.25	17.13	Loss due to remaining organic moiety

**Table 5:** Powder X-ray data of Cu(II) complex.

Peak	2*θ*	*θ*	sin⁡⁡θ	sin⁡^2^⁡θ	1000sin⁡^2^⁡θ	1000sin⁡^2^⁡θ/CF (*h* ^2^ + *k* ^2^ + *l* ^2^)	*h* *k* *l*	*d*		*a* in Å
								Obs.	Calc.	
1	5.758	2.879	0.050	0.0025	2.52	1.00 (1)	(1 0 0)	15.335	15.338	15.330
2	26.804	13.402	0.231	0.0536	53.68	21.301 (21)	(4 2 1)	3.323	3.323	15.338
3	28.790	14.395	0.248	0.0618	61.80	24.523 (25)	(4 3 0)	3.098	3.097	15.338
4	35.480	17.74	0.304	0.0927	92.78	36.817 (37)	(6 1 0)	2.528	2.527	15.338
5	42.777	21.388	0.364	0.1329	132.93	52.750 (53)	(6 4 1)	2.112	2.111	15.338
6	48.302	24.151	0.409	0.1673	167.36	66.412 (66)	(5 5 4)	1.882	1.882	15.338
7	53.771	26.885	0.452	0.2044	204.48	81.142 (81)	(8 4 1)	1.703	1.702	15.338
8	57.486	28.743	0.480	0.2311	231.16	91.733 (92)	(— — —)	1.601	1.601	15.339

**Table 6:** Powder X-ray data of Ni(II) complex.

Peak	2*θ*	*θ*	sin⁡⁡θ	sin⁡^2^⁡θ	1000sin⁡^2^⁡θ	1000sin⁡^2^⁡θ/CF (*h* ^2^ + *k* ^2^ + *l* ^2^)	*h* *k* *l*	*d*		*a* in Å
								Obs.	Calc.	
1	6.099	3.049	0.053	0.0028	2.83	1.00 (1)	(1 0 0)	14.480	14.476	14.474
2	26.903	13.451	0.232	0.0541	54.11	19.120 (19)	(3 3 1)	3.311	3.310	14.475
3	29.603	14.801	0.255	0.0652	65.26	23.061 (23)	(— — —)	3.015	3.014	14.481
4	35.529	17.764	0.305	0.0930	93.08	32.892 (33)	(5 2 2)	2.524	2.523	14.480
5	40.704	20.352	0.347	0.1209	120.95	42.738 (43)	(— — —)	2.214	2.214	14.477
6	42.874	21.437	0.365	0.1335	133.57	47.197 (47)	(— — —)	2.107	2.107	14.477
7	53.747	26.873	0.452	0.2043	204.32	72.196 (72)	(6 6 0)	1.704	1.703	14.474
8	54.889	27.444	0.460	0.2124	212.418	75.057 (75)	(7 5 1)	1.671	1.671	14.474
9	56.054	28.027	0.469	0.2207	220.794	78.016 (78)	(7 5 2)	1.639	1.638	14.477

**Table 7:** Minimum Inhibitory Concentration (MIC in *μ*g/mL) of Schiff base ligand **(L) **and its metal complexes.

Organisms	Concentration (*μ*g/mL)										
	Compounds	0.195	0.39	0.78	1.563	3.125	6.25	12.50	25	50	100
*Enterobacter Aerogenes *	Ligand **(L)**	+	+	+	+	∗∗∗	−	−	−	−	−
	Cu-Complex	+	+	+	∗∗∗	−	−	−	−	−	−
	Co-Complex	+	+	+	∗∗∗	−	−	−	−	−	−
	Ni-Complex	+	+	+	+	∗∗∗	−	−	−	−	−
	Zn-Complex	+	+	+	∗∗∗	−	−	−	−	−	−

*Pseudomonas aerugenosa *	Ligand **(L)**	+	+	+	+	+	∗∗∗	−	−	−	−
	Cu-Complex	+	+	∗∗∗	−	−	−	−	−	−	−
	Co-Complex	+	+	∗∗∗	−	−	−	−	−	−	−
	Ni-Complex	+	+	+	∗∗∗	−	−	−	−	−	−
	Zn-Complex	+	+	∗∗∗	−	−	−	−	−	−	−

*Aspergillus niger *	Ligand **(L)**	+	+	+	+	+	+	∗∗∗	−	−	−
	Cu-Complex	+	+	+	∗∗∗	−	−	−	−	−	−
	Co-Complex	+	+	+	+	+	∗∗∗	−	−	−	−
	Ni-Complex	+	+	+	∗∗∗	−	−	−	−	−	−
	Zn-Complex	+	+	+	+	∗∗∗	−	−	−	−	−

*Aspergillus flavus *	Ligand **(L)**	+	+	+	+	+	∗∗∗	−	−	−	−
	Cu-Complex	+	+	+	+	∗∗∗	−	−	−	−	−
	Co-Complex	+	+	+	+	+	+	+	∗∗∗	−	−
	Ni-Complex	+	+	+	+	∗∗∗	−	−	−	−	−
	Zn-Complex	+	+	+	+	∗∗∗	−	−	−	−	−

^+^indicates turbidity is observed.

^−^indicates turbidity is not observed.

***represents the MIC value.

**Table 8:** Brine shrimp bioassay data of the Schiff base ligand **(L) **and its metal complexes.

Compound	LD_50_ (M/mL)
Ligand **(L)**	2.470 × 10^−4^
Cu-Complex	1.387 × 10^−4^
Co-Complex	1.168 × 10^−4^
Ni-Complex	1.074 × 10^−4^
Zn-Complex	2.308 × 10^−4^
